# Upgrading the Power Grid Functionalities with Broadband Power Line Communications: Basis, Applications, Current Trends and Challenges

**DOI:** 10.3390/s22124348

**Published:** 2022-06-08

**Authors:** Jon González-Ramos, Noelia Uribe-Pérez, Alberto Sendin, David Gil, David de la Vega, Igor Fernández, Ignacio Javier Núñez

**Affiliations:** 1 Bilbao Engineering College, University of the Basque Country (UPV/EHU), 48013 Bilbao, Bizkaia, Spain; david.delavega@ehu.eus (D.d.l.V.); igor.fernandez@ehu.eus (I.F.); 2 TECNALIA, Basque Research and Technology Alliance (BRTA), Parque Científico y Tecnológico de Bizkaia, C/Astondo Bidea, Edificio 700, 48160 Derio, Bizkaia, Spain; noelia.uribe@tecnalia.com (N.U.-P.); javier.nunez@tecnalia.com (I.J.N.); 3 Telecommunications Department, Iberdrola, 48003 Bilbao, Bizkaia, Spain; asendin@iberdrola.es; 4 ZIV Automation, Parque Científico y Tecnológico de Bizkaia 210, 48170 Derio, Bizkaia, Spain; david.gil@zivautomation.com

**Keywords:** Power Line Communications, BPL, Smart Grids, Smart Cities, broadband communications, electrical grid, data transmission, propagation, communication standards, EMI

## Abstract

This article reviews the basis and the main aspects of the recent evolution of Broadband Power Line Communications (BB-PLC or, more commonly, BPL) technologies. The article starts describing the organizations and alliances involved in the development and evolution of BPL systems, as well as the standardization institutions working on PLC technologies. Then, a short description of the technical foundation of the recent proposed technologies and a comparison of the main specifications are presented; the regulatory activities related to the limits of emissions and immunity are also addressed. Finally, some representative applications of BPL and some selected use cases enabled by these technologies are summarized, together with the main challenges to be faced.

## 1. Introduction

Technologies for Power Line Communications (PLC) are the preferred alternative selected by the Distribution System Operators (DSOs) for the data transmission that paves the way for the development of the existing and prospective applications for Smart Grids (SGs). The main advantages of this technology rely on the fact that the grid is already deployed, avoiding the costs of new infrastructures or the dependency of third parties in the data transmission and management [1,2], while enabling Low Voltage (LV) and Medium Voltage (MV) grid automation within the SG concept.

The development of different Narrowband Power Line Communications (NB-PLC) technologies, which have emerged from different international industrial endeavors within several alliances, enabled the deployment of smart metering, the inspection of electrical signal quality, and the remote monitoring of the devices connected to the grid [3,4,5,6]. None of these applications require highly demanding features from the transmission technologies’ perspective. Future SG applications, in contrast, related to the integration of renewable energies, electric vehicle (EV) charging management, and energy demand response [1], among others, will need real-time responses from telecommunications devices, a higher bandwidth, the priority management of the data to be transmitted, in addition to greater cybersecurity requirements. Moreover, these new services will enable the evolution of a fully automated and distributed power grid architecture, where each node can be both a producer and a consumer at the same time, thus facilitating a fast and reliable communications system. Thus, the classical and hierarchical model of production-distribution-consumption will be transformed into a distributed and flexible architecture [7].

In this inexorable evolution of the electrical grid, together with the strict requirements of the new grid services, high-performance communication technologies become essential. For this reason, the DSOs are turning their gaze to Broadband Power Line Communications (BB-PLC or BPL), as these advanced technologies may provide considerably higher data rate transmissions, lower latency times, and an increased communication robustness, in frequencies up to 250 MHz [2] in their MV and LV grids.

This article covers several aspects and perspectives related to the recently developed BPL technologies, with the aim of providing a holistic picture to the technical and scientific community. First, in Section 2 and Section 3, the organizations, alliances, and standardization organisms are outlined. In Section 4, the technical features of the recently proposed technologies are described and compared, whereas the main regulatory directives and standards for the development of BPL equipment are summarized in Section 5. Finally, some of the main applications of BPL in the electricity grid are presented, as well as the challenges that these technologies must face for a successful deployment.

## 2. Organizations and Alliances Involved in the Development of BPL Technologies

BPL implementations were first developed as the result of two driving factors. First, the normal evolution of existing PLC systems, with the intention to provide higher data rates, and second, and more importantly, the application of techniques already present in other telecommunication scenarios, to the grid power lines [8].

Several organizations worldwide have been leading the development of BPL technologies, some of them promoting recommendations and technical standards, and others defining and proposing specific transmission technologies with a market-oriented focus. Broadband Forum is the main organization promoting technical standards, whereas the technologies oriented to the market have been developed (and mostly still are) by HomeGrid Forum, HD-PLC Alliance, HomePlug Alliance, and PRIME Alliance.

### 2.1. Broadband Forum

The Broadband Forum [9] is a communications industry consortium focused on endorsing standards, technical reports, and specifications about broadband technologies. It is an open, non-profit industry organization composed of broadband operators, vendors, consultants, and testing laboratories.

Projects supported by The Broadband Forum cover technologies for Connected Home, broadband (gigabit capable) access network, Software Defined Networking, Network Functions Visualization, and applications of 5G, such as autonomous cars, industrial Internet of Things (IoT), or smart communities [9]. These technologies and applications require specifications for multi-service broadband packet networking and service management. For this purpose, the forum is involved in the development of software data models, reference implementations, testing and certification programs, and specifications for interoperability.

In particular, the Broadband Forum has published, in collaboration with Home Grid Forum, a detailed testbed, together with a set of tests that enable a performance comparison between BPL products and technologies that can be independently verified [10]. Several categories of tests are included in this testbed, such as throughput performances, noise immunity, topology, traffic, security, and Quality of Service (QoS), among others. The testbed is aimed at providing the industry, operators, and test labs with a useful tool to verify a wide range of features for future deployments of BPL technologies.

### 2.2. HomeGrid Forum

HomeGrid Forum [11] is an industry alliance created to support the development and deployment of a specific broadband transmission technology, called Gigabit Home Networking (G.hn), based on standards developed by the International Telecommunication Union (ITU), and described in detail in Section 4.1. HomeGrid Forum complements the activity of ITU to test the G.hn solutions, maintaining a comprehensive compliance and interoperability program to promote an ecosystem of silicon and final products based on the G.hn standards. The focus is on the definition, development, and control of the certification process to ensure the compliance and interoperability of products following the standards. Their activities have been mainly focused on the in-home environment, although last-mile access (specifically multi-dwelling environments) is also addressed.

HomeGrid Forum is making efforts to promote the role of G.hn for the SG evolution and to adapt G.996x, the set of ITU recommendations related to BPL, to specific application scenarios (see next sections). This implies the definition of appropriate configurations to support longer distances, noisy environments, and a larger number of nodes, as well as IEEE 802.1X authentication frameworks, as the latest ITU-T roadmap states [12]. Recently, E.ON joined HomeGrid Forum to further influence the progress of G.hn technology for the energy sector [13].

In addition, HomeGrid Forum issues certification testing through accredited companies. The testing procedure is designed to ensure that commercial products containing both G.hn silicon and production software/firmware comply with ITU-T G.hn standards and the HomeGrid requirements regarding interoperability and performance, as specified in the HomeGrid Forum Certification Test Plans.

HomeGrid Forum collaborated with The Broadband Forum to develop an interoperability program to verify the adherence of commercial products to the ITU standard, by means of a performance test plan [14]. In this activity, the HomeGrid Forum was in charge of applying the formal Compliance and Interoperability program for the ITU standard.

### 2.3. High Definition Power Line Communication (HD-PLC) Alliance

The High Definition Power Line Communication (HD-PLC) Alliance, founded by Panasonic, is a private organization that gathers different actors from the wireline technology segment [15] to spread the use of HD-PLC technology and improve the communication compatibility between products adopting this communication technology. HD-PLC technology supports applications on a large scale, such as building automation and smart factories, so that any existing cabling installation (and specifically power lines) can be turned into a high-speed network.

Some applicable use cases defined by the HD-PLC are smart metering, data transmission in the elevator system, the data collection of measuring instruments in factories, and streetlight management.

### 2.4. HomePlug Alliance

The HomePlug Alliance was founded in 2000, comprising 70 member companies, including Atheros Communications (ATHR), CISCO, and GE Energy, among others. Its main objective was to create specifications and certification programs for using power lines for reliable home networking and SG applications. The alliance enabled and promoted the HomePlug technology in collaboration with international standardization organizations, such as the IEEE, apart from using market development and user education programs [16,17].

HomePlug Powerline Alliance developed other specifications outside the pure broadband domain, namely HomePlug Command and Control specification and HomePlug Green PHY specification, in 2007 and 2010, respectively. The latter is for lower-cost Smart Energy solutions as a subset of HomePlug AV with peak rates up to 10 Mbps [18]. These technologies are described in Section 4.4.

HomePlug alliance stopped its activities in 2016 [19].

### 2.5. PoweRline Intelligent Metering Evolution (PRIME) Alliance

The PoweRline Intelligent Metering Evolution (PRIME) Alliance, promoted by utilities such as Iberdrola, Naturgy, E-REDES, E.ON, Viesgo, and Energa, is focused on the development of an open, public, and non-proprietary telecommunication solution to support not only smart metering functionalities but also network control and monitoring applications [20]. PRIME is an open technology for NB-PLC, standardized by ITU (ITU-T G.9904), for SG in general, and for smart metering in its initial uses. One of the main efforts of the PRIME Alliance was to accomplish interoperability between equipment and systems from different manufacturers. This technology was adopted by several DSOs, which consisted of deployments of tens of millions of smart meters in more than 15 countries in Europe and the Middle East [20].

Beyond NB-PLC, in 2019, the PRIME Alliance created a BPL task force to address the standardization process of the overall architecture of a BPL solution, aiming at an open, interoperable, and broadband PLC solution. For this purpose, several chipset and communication device manufacturers also participate in the PRIME Alliance’s task force. In 2020, the existing alternatives used for in-home BPL, such as HomePlug or G.hn, were analyzed by this task force, to evaluate the adaptation, or the direct adoption, of its use in the electrical distribution grid. Some initial tests suggested that G.hn technology could be a good candidate to be adapted to new scenarios as the basis for the development of a PRIME-BPL technology. Currently, some field tests to evaluate the performance of the G.hn technology in the distribution grids have been carried out in Germany [21].

## 3. PLC Standardization

### 3.1. International Telecommunication Union (ITU)

The ITU is the United Nations agency for information and communication technologies. It allocates global radio spectra, assigns satellite orbits, and develops technical standards so that the interconnection between technologies and networks is ensured, and aims at improving access to common telecommunications infrastructures in order to develop communities worldwide [22]. The ITU-T is the standardization sector within the ITU structure, in charge of defining and implementing global communication standards, except for radio communication services. Different thematic study groups undertake the standardization work. In particular, the ITU-T SG15 (study group 15—“Networks, Technologies and Infrastructures for Transport, Access and Home”) defines the specifications of cable-based data transmission technologies and architectures between devices connected to transport networks or in-home local networks.

The main standard related to BPL published by this study group is under the umbrella of G.hn, a family of standards that defines transmission technologies within home networks, over different types of cable networks (power lines, phone lines, and coaxial cables). The following list gathers the set of recommendations regarding BPL:

- ITU-T Rec. G.9960: Unified high-speed wireline-based home networking transceivers—system architecture and physical layer specification [23];
- ITU-T Rec. G.9961: Unified high-speed wireline-based home networking transceivers—data link layer specification [24];
- ITU-T Rec. G.9962 (G.hn-MGM): Unified high-speed wire-line based home networking transceivers—management specification [25];
- ITU-T Rec. G.9963 (G.MIMO): Unified high-speed wireline-based home networking transceivers—multiple input/multiple output specification [26];
- ITU-T Rec. G.9964 (G.hn-PSD): Unified high-speed wireline-based home networking transceivers—power spectral density specification [27].

In addition, several related recommendations have been published:

- ITU-T Rec. G.9972 (G.cx): coexistence mechanism for wireline home networking transceivers [28];
- ITU-T Rec. G.9977: mitigation of interferences between DSL and PLC [29];
- ITU-T Rec. G.9978: secure admission in a G.hn network [30];
- ITU-T Rec. G.9979: implementation of the generic mechanism in the IEEE 1905.1a-2014 standard to include applicable ITU-T recommendations [31];
- ITU-T Rec. G.9980 (G.cwmp): remote management of customer premises equipment over broadband networks—customer premises equipment WAN management protocol [32].

### 3.2. IEEE ComSoc, Power Line Communications Standards Committee (PLCSC)

The IEEE Communications Society co-sponsors technical standardization work on different communications topics [33]. Within this society, the Power Line Communications Standards Committee (PLCSC) intends to develop and maintain standards on communications over power lines, considering different scenarios such as the distribution grid, microgrid, and in-home or in-vehicle, among others, and applications such as Distributed Energy Resources (DER) management, Advanced Metering Infrastructure (AMI), and the management of Home Area Networks (HAN).

The Broadband Over Power Lines PHY/MAC Working Group, one of the five Working Groups that comprise the committee [34], published the IEEE Standard 1901–2010 “IEEE Standard for Broadband over Power Line Networks”. This standard defines the physical (PHY) and medium access control (MAC) layers of BPL technology for local area networks (LANs), smart energy, SG, IoT, transportation platform (vehicle) applications, and other forms of data distribution [35]. For the development of these applications, the standard proposes the use of transmission frequencies for BPL up to 100 MHz. Eventually, the IEEE Standard 1901 has evolved into the 1901–2020 version, thus expanding the standard’s reach to other applications that will be explained in this article.

The focus of the standard is the balanced and efficient use of the channel by all classes of BPL devices. For this purpose, mechanisms for coexistence and interoperability between different BPL devices are defined, with the aim of ensuring the required minimum bandwidth and the expected quality of service.

Moreover, to provide a heterogeneous PLC-wireless solution to the distribution and in home-area networks, the IEEE 1905.1 standard for a Convergent Digital Home Network for Heterogeneous Technologies, defines an abstraction layer for multiple technologies [36]. This abstraction layer ensures that upper layer protocols do not depend on the characteristics of the underlying technologies.

In addition, the PLCSC published a complementary standard for the lowest frequency range assigned to BPL (1.6 MHz to 12 MHz), as this narrower frequency range simplifies the design and implementation of the transmission devices, while still providing broadband services [37]. Moreover, the “IEEE 1901.1 for Medium Frequency (less than 15 MHz) Power Line Communications for Smart Grid Applications” [38] specifies test procedures for the compliance and interoperability testing of devices implementing the IEEE 1901.1 standard, for this so-called medium frequency band. The document also describes application scenarios and use cases for IEEE 1901.1, associated channel models, and performance expectations; moreover, a collection of potential implementations allowing enhancement of test procedures are included.

### 3.3. International Organization for Standardization/International Electrotechnical Commission (ISO/IEC)

The main contribution of ISO/IEC to the development and standardization of new BPL technologies is included in ISO/IEC 12139-1:2009 [39], where a description of PHY and MAC layers for high-speed power line communication devices is presented. This standard is based on the KS X 4600-1, a Korean broadband power line technology, which is described in Section 4.6.

With the aim of ensuring the coexistence between BPL technologies, specifically between ISO/IEC 12139-1 and ITU-T G.9960, and ISO/IEC 12139-1 and IEEE Std 1901-2010, a mechanism for information exchange was published in ISO/IEC 21228:2019 [40]. Thus, interferences between different standard-based BPL devices are avoided. The main technical details of this report, carried out by the study group on High-Speed PLC Harmonization established by ISO/IEC JTC1/SC 6, are gathered in Section 4.7.

## 4. Fundamentals of BPL Technologies

In this section, the principal characteristics of the more relevant BPL technologies applicable to the LV distribution grid are described. These technologies are the technical basis for enabling the deployment of new functionalities in grid digitalization, such as distribution automation (telecontrol) and AMI.

### 4.1. Gigabit Home Networking (G.hn)

G.hn, standardized by the ITU-T, is a data transmission technology supported and promoted by the HomeGrid Forum Alliance for different propagation media (coaxial, phone line and optical fiber) within home networks, achieving data rates of up to 1 Gbps. Although originally developed for in-home communications, it was identified by some DSOs as an adequate basis for broadband communications in the distribution grid. Therefore, since 2009, they are trying to adapt to other markets such as smart metering [41], SG [42], and industrial networking ones [43].

As mentioned in Section 3.1, the G.hn technology is described by a set of ITU-T standards (recommendations G.9960 to G.9964 [23,24,25,26,27]). G.hn includes the capability to notch specific frequency bands to avoid interference with amateur radio bands and other licensed radio services, as well as mechanisms to avoid interference with legacy home networking technologies.

The PHY layer is based on Orthogonal Frequency Division Multiplexing (OFDM) modulation for a frequency band schedule up to 50 MHz or 100 MHz, and a so-called Low-Complexity Profile (LCP) up to 25 MHz. This LCP is mentioned in [41] when discussing potential Smart Metering scenarios. Additionally, low-density parity-check codes (LDPC) are applied as a technique for forward error correction (FEC) to increase the robustness of the transmission.

The MAC layer in G.hn is based on Time Division Multiple Access (TDMA). The ITU-T SG15 published a Technical Paper in 2019 considering the use of BPL access in SG networks [41,43], and it has recently published a new Technical Paper focused on adapting the technologies described in the ITU-T G.996x standards to the new requirements of SG applications [42]. With this approach, four use cases were identified for the use of G.hn in LV distribution grids: smart metering, smart meter gateway, narrowband smart meter concentrator, and MV backbone. The improvements provided by G.hn technology in these use cases include the support of mesh and tree topologies, the routing functionalities to support a large number of nodes, and the support of the IEEE 802.1X authentication framework and management data models [12].

The Data link layer (DLL) of this technology is defined in Rec. ITU-T G.9961 [24], with the use of contention-free TDMA and contention-based Carrier Sense Multiple Access (CSMA) MAC, among other layer-2 mechanisms. The data management is described in the Rec. ITU-T G.9962 standard [25], whereas in ITU-T G.9963 the application of Multiple Input/Multiple Output (MIMO) procedures is specified [26]. Finally, Rec. ITU-T G.9964 [27] states the Power Spectral Density (PSD) limits for transmission devices as frequencies between 2 MHz and 30 MHz, without referring to other possible local or regional regulations.

### 4.2. IEEE 1901

The IEEE 1901–2010 standard [44], published in December 2010, derives from the work initiated by the IEEE P1901 working group in 2007, that selected a consolidated proposal by the HomePlug Powerline Alliance and the HD-PLC Alliance. Hence, the IEEE 1901 standard is a trade-off solution between the Fast Fourier Transform (FFT)-based OFDM PHY defined for HomePlug AV and the Wavelet-based OFDM PHY used in Panasonic’s HD-PLC devices. Both options for the PHY layer are optional, but not interoperable. To solve this issue, IEEE 1901 defines an InterSystem Protocol (ISP) providing coexistence both among IEEE 1901 incompatible PHYs, but also with other systems (e.g., ITU-T G.hn [28]) and other grid segments (in-home and access). It is remarkable that legacy HomePlug AV and the current IEEE 1901 versions remain incompatible. Additionally, IEEE 1901 defines a Coexistence Protocol (CXP), so that non-IEEE-1901 conformant devices coexist to IEEE-1901-conformant devices or non-IEEE-1901-conformant devices. This standard enables high-speed communications, with data rates up to 100 Mbps, in the frequency range from 1.8 MHz–50 MHz.

In the full FFT-based 1901 mode, a 24.414 kHz carrier spacing is defined. In the operation bandwidth, up to 1974 carriers are employed, modulated with phase shift keying (PSK) modulations; Binary PSK (BPSK); Quadrature PSK (QPSK); 8 Quadrature Amplitude Modulation (8 QAM), 16 QAM, 64 QAM, 256 QAM, 1024 QAM, or 4096 QAM. Moreover, three robust signaling schemes, ROBO-FTT OFDM modes, are also defined.

In contrast, the Single Channel Wavelet (SCW) specification offers robustness against selective frequency channels and narrowband noise, along with providing a very efficient utilization of the spectrum. Wavelet OFDM frames can be directly transmitted at baseband or modulated to a specific carrier. In the case of in-home and access applications, baseband transmission is mandatory, whereas modulated transmission is optional. For each operation mode, different characteristics are defined.

The baseband PHY utilizes 512 equally spaced carriers in the frequency band from 0 Hz to 31.25 MHz. This standard uses the frequency range from 1.8–28 MHz with an optional band of 30–50 MHz. Each carrier is modulated with 2-Pulse Amplitude Modulation (PAM), 4-PAM, 8-PAM, 16-PAM, or 32-PAM, when operating in the high-speed mode. The diversity mode, in contrast, uses 2-PAM modulation and further frequency diversity, so that the PHY layer can operate under adverse conditions. This Wavelet PHY layer includes Reed Solomon, convolutional Viterbi, or LDPC-CC encoders.

The modulated SCW PHY uses the same FEC and modulation schemes described in the baseband PHY to transmit the data through 1024 carriers in any band within the frequency range from 1.8–50 MHz.

Regarding the MAC layer, two types of service can be provided: connection-oriented services or connectionless services. In both cases, a TDMA scheme, providing a contention-free period, and a CSMA / Collision Avoidance (CSMA/CA) scheme, in which a contention period is provided, can be used.

In September 2020, a revision of the standard was carried out, publishing the IEEE 1901–2020 [35], where a Flexible Channel Wavelet (FCW) PHY option is included. This way, changes in the operation modes in different channels with different carrier spacing values are allowed. This enables high-speed and long-distance communications, which are considerably required in the currently highly demanded IoT applications. This PHY layer only considers frame baseband transmissions. Although, in general, it includes similar characteristics to the SCW PHY, it allows optional transmissions in the 31.25 MHz–62.5 MHz frequency range when the sampling rate is doubled, and up to 100 MHz if the sampling rate is multiplied by a factor of four. In addition, as wireless communication, it enables a plurality of channels, due to the subdivision into two or four channels provided by this Wavelet-based PHY layer.

### 4.3. HD-PLC

HD-PLC technology is structured in four generations [45]. The first two generations were designed to achieve a maximum data throughput of 190 Mbps and 210 Mbps, for transmissions in the frequency range from 4 MHz to 28 MHz and 2 MHz to 28 MHz, respectively; nevertheless, they did not end up in a standard.

The third generation was divided into two specifications: HD-PLC3 Complete and HD-PLC3 Muti-hop, applicable to different scenarios. HD-PLC3 Complete is based on the IEEE 1901–2010 communication standard, adopting the Wavelet-based PHY layer, which aims at accomplishing a maximum data transmission rate of 240 Mbps. Thus, while FFT-OFDM is mostly found in Europe and the United States, Wavelet-OFDM modulation is most common in Japan.

HD-PLC3 Multi-Hop includes hopping capabilities between network nodes, to overcome one-to-one communication capabilities. This version of HD-PLC refers to the IEEE 1901–2010 standard, combined with the ITU-T G.9905 standard. It is based on a so-called Centralized Metric-Based Source Routing (CMSR) protocol, which allows the data signal to progress among terminals connected to one master node in a tree-like structure. The maximum number of hops is limited to 10, in a network of up to 1024 nodes. The network topology is the main difference between the Multi-hop and the Complete Standards.

In the fourth generation (HD-PLC4) [46], the IEEE 1901–2010 standard was adapted to from IEEE 1901a–2019, including both the so-called FCW to accommodate smaller bandwidth channels (typical of access scenarios) and the option of an extended band (from 31.25 MHz to 62.5 MHz). The FCW offers 15 selectable channels; options considering wider bandwidths expand the data transmission rate up to a maximum of 1 Gbps, while others with smaller bandwidths may result in longer ranges (estimated up to 2.5 times with respect to IEEE 1901–2010).

The HD-PLC4 also considers an extended ISP (E-ISP), which was adapted according to the new features of other PLC systems in the market. This functionality was eventually consolidated in the IEEE 1901–2020. For a general robustness, the PHY layer includes the use of several forward error correction techniques, such as Reed–Solomon, Convolutional Codes, and LDPC codes [46].

### 4.4. HomePlug

The HomePlug 1.0 specification was published in June 2001 [47] and HomePlug 1.0.1 [48] in December 2001. This technology achieved a 14 Mbps maximum data throughput and it was eventually adopted as the TIA-1113 international standard within the subcommittee TR-30.1 of the Telecommunications Industry Association [49]. The activity in HomePlug continued with the HomePlug AV specification [50], which describes a PLC system operating at 200 Mbps. The PHY layer is based on FFT-OFDM multiplexing, in the frequency band of 2 MHz to 28 MHz, with modulation up to 1024 QAM and turbo convolutional codes as an FEC. The MAC protocol is a hybrid configuration of TDMA/CSMA/CA, similar to the protocol adopted by HomePlug 1.0.1.

In June of 2010, the HomePlug Green PHY specification [18] was published as a derivative of HomePlug AV. It uses the frequency band from 2 MHz to 30 MHz, allowing interoperability with HomePlug AV and IEEE P1901. In contrast to these previous systems, it only supports QPSK modulation with turbo convolutional code as an FEC. This restriction, together with the restrictive data rates supported by ROBO modes, limits the maximum bitrate to 10 Mbps [18].

The HomePlug AV2 specification was introduced in 2012 as HomePlug AV2 2.0 [51]. It claims to be interoperable with HomePlug AV and HomePlug GreenPHY devices, and it supports MIMO and a wider frequency band (1.8 MHz–86.13 MHz) to offer up to 1.5 Gbps PHY data rate throughput. HomePlug AV2 2.1 followed in February 2014 [52].

### 4.5. Open PLC European Research Alliance (OPERA)

A system based on Open PLC European Research Alliance (OPERA) specifications and applicable to different grids’ access scenarios was released in 2006 [53] and applied to SGs [54]. OPERA is the name for a European Commission-funded research and development project [55].

OPERA specifications [56,57] were mainly in the form of a PHY layer and a MAC layer. The PHY is an OFDM-based signal, with the purpose of achieving a maximum data rate of 200 Mbps. The PHY uses up to 1536 subcarriers with configurable bandwidths of 10 MHz, 20 MHz, or 30 MHz, and Amplitude Differential PSK(ADPSK) with up to 1024 points per constellation. Additional features include adaptive modulation, frequency notching, Reed–Solomon block codes-based FEC, and truncated four-dimensional Trellis coded modulation.

The MAC layer is a TDMA-based layer, where a time slot for transmission is assigned to each device. A head-end device, acting as a master, controls the use of channel resources to guarantee QoS, in terms of throughput and network latency.

The Universal Powerline Association (UPA), an international association working to promote global standards and regulations, collaborated and promoted this access standard [58,59], although it eventually became aligned with HomeGrid Forum [42].

### 4.6. KS X 4600-1

The ISO/IEC 12139-1:2009 [39] is a Korean system, initially defined as KS X 4600-1, and later adopted by ISO and IEC in 2009. The standard defines both PHY and MAC layers to support both IEEE 802.3 and serial protocols, using frequencies below 30 MHz.

The PHY uses a Discrete Multi-Tone (DMT) method in frequencies between 2.15 MHz and 23.15 MHz with a subcarrier spacing of 97.65625 kHz (25 MHz/256), and adaptive modulation of differential BPSK (DBPSK), DQPSK, or D8PSK, using Reed–Solomon and convolutional coding as FEC mechanisms.

The MAC layer uses CSMA/CA as access technique and IEEE 802.3 MAC addresses.

### 4.7. Coexistence between BPL Technologies

As briefly mentioned in Section 3.3, a coexistence mechanism between broadband power line technologies that was intended to avoid interferences between different standard-based BPL systems was standardized in 2019 [40]. This mechanism, based on Time Domain Multiplex (TDM) and Frequency Domain Multiplex (FDM), allows up to four non-interoperable communication systems to share time and frequency resources at a time.

Two different Extended TDM resource allocations are described in this international standard: the Extended TDM resource allocation, utilizing resource for an absent in-home system, and the Extended TDM resource allocation utilizing the resource for access system. The first of them extends the TDM general resource allocation map, defined in the IEEE 1901–2010 standard. Hence, from a situation where the in-home-W (IH-W), IH-O, and IH-G access systems are supported, another non-interoperable in-home system, IH-A, is included (the absence of one of the abovementioned systems is necessary for the use of this extended allocation). The mechanism is based on assigning the resources allocated to the first absent in-home system (in the order IH-W, IH-O, and IH-G) to the IH-A. In contrast, the option of the Extended TDM resource allocation is only allowed when there is no access system and no absent in-home system. In this case, the mechanism also begins with the TDM general allocation map; however, the TDMs of the access system are assigned to IH-A.

### 4.8. Comparison of BPL Technologies

To facilitate the direct comparison of the BPL technologies described in previous sections, the technical specifications have been compiled and sorted in Table 1.

Table 1 shows the technological evolution of the proposed standards, as the latest versions of each technology are a forward leap in the technical features. This is accomplished with the use of high-order modulation schemes with a high number of symbols, achieving data rates up to 1.5 Gbps and 1 Gbps for HomePlug AV 2.0 and the 4th version of HD-PLC, respectively. It is noticeable that different bandwidths are proposed by all the technologies, which is directly related to the available data rate.

All the technologies shown in Table 1 use FEC techniques to increase the robustness of the communications by including redundant information at the transmitter and applying error detection and correction algorithms at the receiver end. As the electrical grid is a harsh propagation medium and the transmission losses are high in these frequency ranges, these coding techniques have become essential for the proper performance of BPL technologies. The most used codes include turbo codes, convolutional codes (bit level), Reed–Solomon (symbol level), and LDPC techniques. The latter have a wide range of configurations, reaching the highest levels of robustness.

In addition, these technologies rely on multicarrier modulation schemes based on orthogonal frequency subcarriers (OFDM), which can be generated by Fourier analysis or wavelet implementation.

Finally, it should be noted that TDMA and CSMA/CA are the medium access techniques selected by the BPL technologies. Therefore, the proper development of the technologies requires a high level of synchronization for data transmission between nearby communication devices.

## 5. Regulatory Activities

**Figure 1:**
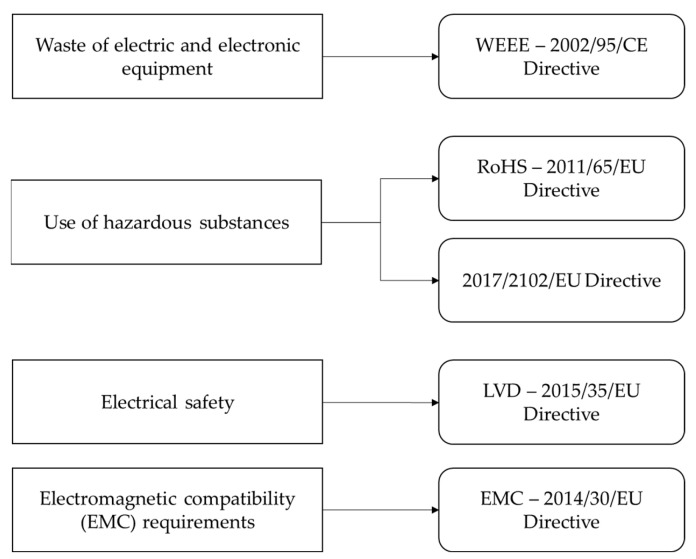
Directives of general application to be considered in the development of BPL electronic devices.

This section summarizes the main regulatory directives and standards to be taken into consideration in the development of BPL equipment. In Figure 1, the directives of general application, briefly described in Section 5.1, are introduced.

**Figure 2:**
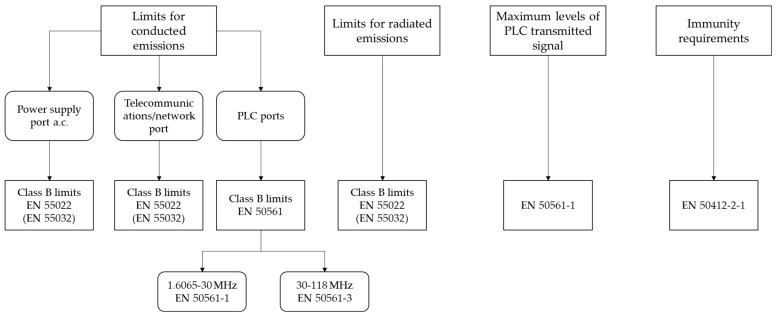
Limits for conducted and radiated emissions, PLC transmitted signal maximum levels, and immunity requirements.

Figure 2 gathers the standards related to the limits for conducted and radiated emissions, as well as the maximum levels specified for the PLC transmitted signal and the immunity requirements for communication devices. The fundamentals of these standards are described in Section 5.2 to Section 5.5.

### 5.1. Directives of General Application

The European Union has defined several directives about the commercialization of electronic products. Most of these directives are of general application and their requirements do not have specific implications for BPL products. This is the case for the regulation about the waste of electric and electronic equipment (WEEE—2002/95/CE Directive) [60], the restriction of the use of hazardous substances to protect the environment and public health (RoHS—2011/65/EU [61] and 2017/2102/EU Directives [62]), and the requirements for electrical safety (LVD—2014/35/EU Directive) [63]. Additionally, the directive EMC 2014/30/EU Directive [64], related to electromagnetic compatibility (EMC) requirements, outlines several aspects to be considered in the development of BPL electronic devices.

### 5.2. Limits for Conducted Disturbance Emissions

Figure 2 shows that different limits for conducted disturbances are defined by the regulation for the power supply, telecommunications/network, and PLC ports. According to the approach of this article, only the requirements related to the PLC ports are described in this section.

The European technical standard EN 50561-1:2013+AC:2015 [65] develops the EMC requirements and it specifies the limits and the measurement methods of the conducted disturbance characteristics in the PLC ports for in-home communication apparatus that use the LV power installation as the transmission medium. This standard is applicable to equipment that communicates over this medium in the frequency range from 1.6065 MHz to 30 MHz. The document EN 50561-3:2016 [66] covers the same items for equipment that communicates using BPL in a frequency range above 30 MHz (typically between 30 MHz and 118 MHz), although this standard is still not included in the list of harmonized standards of the EMC Directive.

Unsymmetrical emissions should be measured using an Artificial Mains Network (AMN), which provides a specific access impedance value at the point of measurement of the terminal voltage, and Line Impedance Stabilization Networks (LISN), with the same purpose but for communication ports.

In the frequency range from 1.6065–30 MHz, quasi-peak and average detectors are employed to evaluate disturbance levels and CISPR 11:2015 [67] class B limits, defined for devices suitable for use in residential areas, are also applied. These limits, which vary considerably depending on the detector used and the frequency range of operation, are shown in Table 2 according to [65].

In the frequency range from 30 MHz to 118 MHz, in contrast, only a peak detector is employed to assess the maximum unsymmetrical conducted emissions in the PLC port. These limits are gathered in [66].

### 5.3. Limits for Radiated Disturbance Emissions

According to [67], no limits are specified for the radiated disturbances in the frequency range from 150 kHz to 30 MHz. However, for the frequencies above 30 MHz, radiated emission limits apply with no differences concerning general EMI standards. Specifically, the class B limits of the European Standard EN 55032 [68] are applicable. These limits, from 30 MHz to 1 GHz, are gathered in Table 3.

As it is shown in the table, depending on the measurement equipment, Open Area Test Site/Semi Anechoic Chamber (OATS/SAC) or Fully Anechoic Chamber (FAR), and the distance, 3 or 10 m, different class B limits are specified. In all cases, a Quasi-peak detector with a 120 kHz bandwidth is applied.

### 5.4. Limits for PLC Transmitted Signals

The European Standard EN 50561-1 also establishes the maximum levels of the transmitted PLC signal in the frequency band 1.6065–30 MHz. These limits are shown in Table 4. The detectors should comply with the requirements stated in the EN 55016-1-1 [65].

It should be mentioned that the PLC port of the BPL devices must additionally incorporate a dynamic power control function to minimize the probability of radio disturbance whilst still maintaining communication. The dynamic power control function should be capable of reducing the output power to the maximum levels given in the EN 50161-1 standard.

### 5.5. Immunity Requirements

Concerning the immunity requirements for communication devices over LV installations in the frequency range from 1.6–30 MHz, the applicable standard, derived from the EMC Directive, is EN 50412-2-1:2005 CORR: 2009 [69]. This includes the following requisites classified on a “type of port” basis:

- Power-frequency magnetic field of 50 Hz;
- Radio-frequency electromagnetic field amplitude modulated;
- Electrostatic discharge;
- Radio-frequency continuous conducted common mode;
- Voltage dips and interrupts;
- Surges;
- Electrical fast transients.

## 6. Applications of BPL

Future uses and functionalities of SGs involving BPL technologies will demand more challenging requirements in communications reliability (link availability and robustness) and performance (data throughput, latency, and bandwidth). In this section, some of the first pilot projects and applications of BPL in the electricity grid are described. The section is not intended to be a detailed compilation of the current applications of BPL for SGs but a description of representative use cases of these technologies.

### 6.1. Smart Metering

Four different use cases related to Smart Metering based on the specifications of BPL technologies can be outlined [42]:

- A Smart Meter (SM) using BPL communication. In this case, the SM integrates the BPL functions for communicating with external data concentrators or head end systems. Hence, BPL technology enables high bandwidth for the smart meter’s communication, allowing Internet Protocol (IP) stack integration. Given the possibilities of the BPL technology, the meter can also encapsulate the traffic of water and gas meters, which can be connected through wireless M-Bus or serial local links to the SM. In this scenario, the AMI infrastructure is fully based on BPL technology, based on the SM.
- A Smart Meter Gateway. In some scenarios, instead of integrating the BPL transmitter/receiver inside the SM, a device called a gateway behaves as a BPL node of the network. The gateway is connected to the SM through its serial connection, and it is in charge of encapsulating the metering traffic into the BPL network. This approach has the advantage of differentiating the devices of metrology and communication infrastructures in separated modules. The gateway may also behave as an intelligent device to connect water meters, gas meters, in-home displays, or even dynamic charges.
- A BPL concentrator of NB-PLC SMs. NB-PLC technology has been proven to be effective at deploying smart metering infrastructures. Nevertheless, as they are low data rate shared transmission networks, in dense environments, the enhanced capacities of BPL can provide an important performance boost with respect to NB-PLC. A reduction in the number of nodes (the number of NB-PLC devices) of each NB-PLC subnetwork provides an effective communication speed-up, as the bandwidth is shared by a reduced number of nodes. Then, a special Gateway encapsulates the NB-PLC traffic into BPL within each centralization node, to provide connectivity to an external data concentrator or head-end system.
- A Smart Metering MV BPL backbone. Once the SM data reach the secondary substation, the backbone connection of this secondary substation can be developed by means of BPL through the MV lines [70,71].

Recently, E.ON is promoting the use of G.hn technology for the energy sector [13], by means of Smart Meter Gateways with G.hn technology to connect energy services central offices with the smart meters located in the users’ homes. The first field trials to test the technology have been presented in [72], where different grid topologies and cable section lengths have been considered.

### 6.2. Grid Automation

The grid automation concept consists of applying automated actions to allow a continuous power supply under circumstances that may cause grid failure. In a centralized grid operation context, it is based on the continuous supervision of the state of the grid via specific equipment (sensors) and the analysis of this information by one or more control centers through SCADA (Supervisory Control and Data Acquisition) systems, which may eventually send commands to grid actuators to reconfigure the grid.

Based on the specifications of BPL technologies, several use cases related to Grid automation can be outlined [73,74,75]:

- Communications with Remote Terminal Units (RTU). The use of BPL in MV lines for SCADA access to RTUs has been used in environments where the use of other communication technologies was not feasible, mainly as a complementary communication technology for access to RTU equipment in remote areas. There are implementations using the 100 kHz–1 MHz frequency range with Spread Spectrum modulation. Later, applications moved to the traditional range from 2 MHz to 25–30 MHz, with OFDM modulations. There are also some implementations based on narrowband G3 PLC technology [73] or HomePlug [75].
- The collection of measurements from grid sensors to control centers or adjacent substations. With the growing deployment of renewable energy sources, it has become necessary to transfer greater amounts of information between the DERs, e.g., PV or wind energy plants, and the substation, to control the injection of energy into the grid. The use of a higher number of sensors, to obtain a more complete and detailed information, demands communication technologies that allow higher data rates and lower latency times. BPL technologies are a good option for such scenarios.
- Energy Control. The use of BPL for the control and monitoring of solar panels has also been applied, mainly for the remote control of the panel tilt to maximize the sun exposure. Real-time monitoring also enables maintenance monitoring, the detection of silicon degradation/need for cell replacement, weather conditions, theft detection, and power output/efficiency.

### 6.3. Electric Vehicle (EV)

The standard ISO 15118 [76,77] specifies a digital communication system between an EV and a charging station, with the purpose of securing the information exchange. It includes functionalities for automatic user authorization without requiring the driver to interact with the charging station, also referred to as Plug and Charge (PnC). It also provides a load management service, based on power schedules and tariff tables. For this purpose, the standard defines specifications for communication, automated authentication, and authorization.

Four use cases are supported by ISO 15118: PnC, smart charging, bidirectional charging, and wireless charging [76,77,78]. Smart charging is based on bidirectional communication between the EV and the charger to support load management. The standard enables load control for variable charging and the charging management of multiple EVs. Several EV manufacturers (Audi, Daimler, Ford, Lucid, Porsche, and VW) are now supporting ISO 151118 PnC [78].

Regarding communications, ISO 15118 defines, firstly, the use cases and requirements for the network, and secondly, the application protocols and the physical and data link layers, not only for wired interfaces [76,79,80], but also for wireless interfaces [77,81,82]. With respect to authentication, the standards describe how to exchange digital certificates to ensure secure communication. This standard is a step further in the communication between the charger and the EV with far greater capabilities than the DIN SPEC 70121 [83], which provides a Pulse Width Modulation (PWM) signal to negotiate the status of the charge and power management.

Beyond all these functionalities, BPL is a transmission technology that may fulfil the requirements of many other use cases in the EV charging infrastructure, which could be more demanding than the point-to-point communication between the EV and the charging point for authentication and payment. In particular, when there are several EV chargers in the same parking space, they could communicate through new BPL technologies to behave as a collaborative community of consumers with a common power management system. Additionally, the Vehicle to Grid (V2G) functionality uses the energy storage of the EV as a DER, and this operation requires advanced automation and power exchange control functions. Moreover, BPL technologies could enable the communication between EVs as a LAN to route the data into the communication backbone. This is particularly useful for underground situations, where the lack of coverage hinders or even halts the communications of each EV charger with the rest of the grid.

### 6.4. Distributed Generation

Distributed generation is composed of small power generation equipment connected to a distribution system, with the purpose of meeting local peak loads and/or displacing the need to build additional (or upgrade) local distribution infrastructure [84], even operating in parallel to the distribution system [85]. A DER is any electric power source, including both power generators and electricity storage systems, that is not directly connected to a bulk power system and capable of providing power to cover local needs or exporting active power to the electric system. Hence, DERs refer to solar PV panels, small wind turbines, natural-gas-fired, fuel cells, biomass combustion systems, waste incineration systems, and even EVs, that may be installed to solve local energy needs, but also connected to the distribution grid as distributed generation sources.

The role of DERs in the electric power system is increasingly relevant and needs to be properly integrated into the grid in operational and regulatory terms. The management of distributed generation is a particular case of grid automation, which must consider aspects related to the interconnection and bidirectional power flow between the DERs and the power grid. As these elements are progressively integrated in the LV grid, the presence of reliable and highly performing telecommunications connectivity is not guaranteed, and thus BPL appears to be a very convenient solution [57].

The IEEE 1547-2018 [86] was defined with the purpose of defining the technical specifications for the interconnection and interoperability between electric systems and DERs, including testing procedures. Recently, a use guide of IEEE 1547 has been published [87], which focused on the interconnection of storage DERs with the electrical grid, such as EV charging stations with V2G ability or Uninterruptible Power Supply (UPS) for off-grid use.

The interoperability defined in this standard consists of the capability of exchanging information in a secure and effective way between systems, devices, or applications involved in distributed generation. For this purpose, an interface that allows for communication with the DSO is a mandatory requirement for a specific DER, supporting one of these communication protocols: IEEE 2030.5 [88], IEEE 1815 [89], or SunSpec Modbus [90]. The exchanged information should provide data about DER characteristics, monitoring (operating conditions), configuration (including the ability to perform specific functions), and management (mode settings) [91]. The longest response time (maximum amount of time to respond to the read requests) determined by the standard is 30 s, and DER communicating interfaces should be continuously operating. Nevertheless, the decision to use the local DER communications interface, or by contrast, to incorporate it within a more general communications system, will be determined by the DSO.

### 6.5. Smart City Services

The concept of a Smart City aims at reconverting cities and urban spaces towards more sustainable, accessible, efficient, and inclusive places. In this context, information and communication technologies are a key tool for the development of collaborative businesses, advanced technologies, and new methods of consumption and networking [92].

Beyond traditional power grid services, BPL technologies can enable a wide range of services under the scope of the Smart City concept, since most of the assets that could be managed through BPL are already connected to the power grid (lights and traffic signaling) or potentially close to power sockets (parking and traffic flow). In these scenarios, BPL would provide a fast response time and high data rates, in contrast to some wireless low-cost solutions.

**Figure 3:**
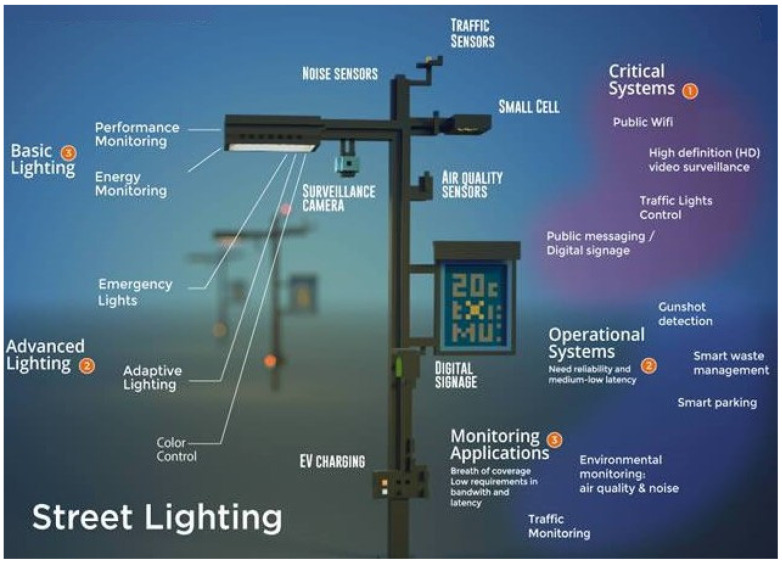
Smart Street Lighting as a Smart City Platform [94].

A representative example is the PLC-based management of urban lighting, already developed in some European cities. Based on this application, the power lines would act as the backbone for networked communications. Additionally, different types of sensors and control equipment could be installed for different purposes other than lighting (traffic monitoring and traffic light management, smart parking, waste management, and the fast signaling of urban services) and connected over the power lines. Then, the information could be transferred to a data/control center to be managed [93]. This way, the outdoor lighting networks could be transformed into BPL high-speed data networks for smart city services, such as video surveillance systems, sensor networks, Wi-Fi access points, and even EV charging stations [94]. Figure 3 shows the Smart City Lighting as a Smart City Platform for multiple services.

Some of the strong points of BPL with respect to other incoming technologies regarding future services in Smart Cities are the non-dependency of batteries, the performance of hundreds of Mbps, and response times of less than 50 ms [95]. Some pilot projects for BPL-based new functionalities related to power control and management have already been implemented. For instance, in the Smart City of Mannheim (Germany), which has integrated both renewable energy and energy storage into the grid through the use of BPL [95].

Another interesting alternative is the hybridization of BPL with RF mesh, which allows for the flexibility of selecting wired or wireless options depending on link availability, channel propagation conditions, or priority algorithms based on data types. Related experiences, such as the deployment of PLC-RF Gateways in the city of Bellingham, Washington, have explored the benefits of the integration of power line and wireless technologies to enable lighting systems to mix and match connectivity options managed through a single central management software [93].

### 6.6. Industrial Applications

The industry 4.0 is the evolution of product mechanization (Industry 1.0), the introduction of the electrification of mass production and assembly lines (Industry 2.0), and the adoption of automation, computers, and electronics (Industry 3.0), as a result of the adoption of communication technologies and the analysis of massive data. Five technologies can be considered disruptive for Industry 4.0 [96]: cyber-physical systems, pervasive connectivity, big data processing, advanced analytics, and smart applications and services.

The rollout of this approach must be supported by a broadband network able to provide high-speed (low latency), reliable (high robustness), and efficient (advanced coding and modulation schemes) data exchange. Additionally, the requirements for an effective broadband infrastructure in Industry 4.0 are simplicity, scalability, security, availability, and affordability [97]. In this challenging context, with high-demanding requirements, BPL technologies can play a decisive role.

The potential targets for the application of BPL technologies within the industrial context are, firstly, those demanding high data rates, such as surveillance and monitoring applications, video IP systems, and interactive panels, as well as internet service for the industry; secondly, those that require very low latency times, such as safety processes or manufacturing procedures; and lastly, communications in extremely noisy environments.

### 6.7. IoT Services

In many aspects, BPL in the IoT follows the same approach as in industry applications, but with a lower cost. According to a recent communication, the market for BPL in the industrial IoT (IoT) context could be more than 350 million ports in 2023 [98]. Wired deployments could be in the form of BPL-based data backbones, which deliver high-speed data to local networks that connect to IoT end-point devices through BPL, other wired assets (e.g., RS-485), or wireless technologies.

The ITU is addressing the adoption of BPL within the IoT paradigm through a specific working group (SG15). This working group is adapting the G.hn standard to IoT, under the name of G.iot, with the purpose of being fully interoperable with G.hn, as part of the same domain and with several coexistence mechanisms [99,100]. This proposal is being developed and aims at addressing domotics and industrial scenarios.

The objectives of this application are [94]:

- Low cost and low consumption, in line with IoT-wireless approaches;
- Low complexity, that is, easy deployments;
- Noise immunity, which is especially important in industrial sites or in locations with high levels of interference;
- Reliability, to guarantee communications at any time;
- Very low value latency and jitter, increasing the availability and allowance of a very high number of nodes, and therefore maximizing the number of potential connected assets.

In addition, IEEE has recently developed the IEEE 1901.3 Standard for Power Line Communications for Internet of Things Applications (IoTPLC), which specifies both PHY and MAC layers. The IoTPLC standard uses wavelet OFDM, which presents better robustness against noise, and it allows higher data rates [101].

Another approach is the 6LoPLC, which adopts a PLC PHY and exploits MAC features of IEEE 802.15.4 devices, as well as 6LoWPAN. In contrast to previously commented solutions, 6LoPLC delivers low-rate PLC [102].

### 6.8. Grid Monitoring

The PLC signals are affected by the grid topology, the anomalies in the grid, and in general, by the performance of the distribution grid as a propagation medium [103]. As a result, PLC devices have been considered by several authors as a good tool to monitor the status of the LV distribution grid. For instance, in [104,105], the two-way handshake of the communications is employed to acquire data of the grid topology. Similar information is obtained by [106] from the grid impedance. In addition, the channel transfer function (CTF) is used in [107] for fault detection, whereas in [108,109] it is used for monitoring the aging of the grid cable. The variations of the CTF are studied in [110] to detect anomalies in the grid used by comparing the CTF properties before and after an occurrence. In the same line, [111] analyzes the variability of the CTF of the reflected signal.

In recent years, some machine learning techniques have been used for grid sensing. As an example, the line impedance, the reflection coefficient, and the CTF are employed for detecting anomalies in [103] using these techniques. The authors of [112] present a model based on machine learning to obtain channel information by means of the S parameters of a cable section, so that cable diagnostic solutions can be performed. The authors of [113] propose algorithms to detect and identify grid faults autonomously that can be implemented in PLC devices.

A potential use beyond grid monitoring that is being investigated is intruder detection. It consists of analyzing variations of the CTF to detect grid intrusions [114,115]. As the CTF is dependent on the physical characteristics of the power line (line length, network topology, and the connected loads [116]), changes in any of these parameters can be used to detect and locate an intruder [117]. Since not all changes in PLC CSIs might be caused by intruders, some techniques such as machine learning can help to differentiate the cause of the change [118,119], and the use of neural networks can also help to improve the security diagnostics [120].

## 7. Challenges for BPL

The main challenges that the BPL technologies must face are the limitations of the electrical grid as a propagation medium for data transmission and the vulnerability of the transmitted data to privacy safeguarding or external attacks.

### 7.1. Limitations of the Propagation Medium

The behavior of the LV distribution grid for data communication is widely unknown for its frequency range of 1 MHz–30 MHz. However, it is already known that the electrical grid is a harsh transmission medium for currently deployed NB-PLC technologies [2,121,122,123], with frequencies up to 150 kHz in Europe and up to 500 kHz in Asia and some countries in the Americas [124,125]. These limitations are associated with the following aspects: grid impedance, transmission losses, resonance effects, and conducted noise and non-intentional emissions (NIE), and some of them are expected to be critical in the frequency range used by BPL technologies in the LV grid. In particular, the transmission losses in this frequency range are expected to be considerably higher than in frequencies assigned to current PLC services (below 500 kHz), and the influence that the loads and the electronic devices connected to the grid have on the propagation phenomenon are still unknown.

The lack of knowledge on power grid performance in this frequency range is due, first, to the absence of models that represent the performance of the LV grid in these frequencies, and second, to the reduced number of field tests developed in the past few years that provide empirical results from measurements in the grid. This last aspect is caused, mainly, by the complexity in developing accurate and reliable measurement systems for this broad frequency range that are also adapted to the harsh conditions of measurements in the grid. In the last few years, several measurement campaigns have been carried out to characterize the power grid properties for frequencies up to 150 kHz, and a few of them have considered frequencies up to 500 kHz. However, for frequencies over 500 kHz, only few field tests have been recently developed. In summary, the features that describe the propagation conditions of broadband transmissions through the electrical grid are poorly known; therefore, the performance of the grid for the frequencies used for BPL technologies can only be estimated by analyzing the tendencies found for frequencies up to 500 kHz.

### 7.2. Grid Impedance

In the frequency range below 9 kHz, the grid impedance is a significant factor for setting EMC requirements. For this reason, reference values of the grid impedance are provided by regulatory bodies for the fundamental frequency (IEC/TR 60725 [126]) and for the frequency range from 2–9 kHz (IEC 61000-4-7 [127]). By contrast, the knowledge about the access impedance and load impedances of LV grids for frequencies above 9 kHz is limited. However, many EMC aspects are affected by the frequency-dependent grid impedance values. First, the line impedance determines the propagation of NIE, and consequently, the potentially disturbing effects on communications [121]. Second, the impedance is also crucial for PLC, as it influences the propagation of the signals. Additionally, the impedance mismatch between communication devices and propagation media limits the power transfer. As an example, very low grid impedance values imply that the transmitter must inject very high current values to transfer appreciable voltage values to reach the receiver [128].

The grid impedance values depend entirely on the grid topology, that is, the number of branches and the length of the cable sections in a particular location, and on the number and type of devices connected at a specific moment. Consequently, the impedance values may change significantly over time [2], which hinders the acquirement of representative impedance values. Traditionally, only long-term impedance variations, mainly due to the connection or disconnection of the electric devices connected to the power grid, have been considered. Nevertheless, the existence of remarkable short-term impedance variations (caused by inverters hosted in some electronic devices that correspond periodically with the fundamental period of the mains signal) has been demonstrated in some studies [129,130].

Previous aspects underline that the characterization of the impedance and the identification of typical impedance values for different frequency ranges are relevant research topics to be addressed for the development of broadband transmission technologies. This characterization must be developed through extensive field trials in various scenarios to develop a statistical approach to obtaining typical impedance values [121,126].

The development of new measurement methods and systems adapted to the harsh conditions of measurements in the LV grid, covering a wider frequency range (up to 500 kHz), and with enhanced accuracy, has been one of the significant contributions in this area in the recent years [131]. Moreover, the definition and development of a metrologically traceable grid impedance standard is a crucial point that enables the objective comparison of the proposed measuring techniques [132].

Some laboratory and field measurements have been developed in recent years, in frequencies up to 150 kHz [133,134,135,136,137], and even up to 500 kHz [136], but there is still a lack of knowledge about the variety of the values in different grid topologies and the user density. For frequencies up to 30 MHz, some values of grid impedance are proposed in [137], based on results for LV previously published in [135,138], although these values are based on a reduced number of measurements.

Results from field trials show impedance values of a few Ohms for frequencies up to 150 kHz and of several tens of Ohms for the frequency range from 150 kHz–500 kHz, with high variability between the different measurement points, as shown in [133,134,135,136]. The typical values proposed in [137] are of a few Ohms for frequencies up to 500 kHz, but they exceed 100 Ohms for frequencies between 8 MHz and 400 MHz. These values provide a reference for the great differences found for different topologies, mainly in the higher frequencies for BPL, and highlight the need to characterize different representative scenarios and different grid topologies.

Lastly, resonant effects have been identified in measurement trials up to 500 kHz, which imply very high impedance values for a narrow bandwidth [136]. Then, the propagation at such frequency ranges is altered, because impedance mismatching situations arise, and then the data transmission can be jeopardized. These resonant effects occur at different frequencies, depending on the grid topology, the length of the cable sections close to the measurement point, and the loads connected in the proximities. They can also change over time. The communication devices could identify resonant effects by employing techniques such as channel sounding and overcome their impact by using different transmission frequencies (frequency agility or frequency diversity); however, the integration of these techniques adds complexity to the communication devices. Resonant effects are also expected in frequencies above 1 MHz, so they could also affect BPL transmission. Nevertheless, the number of occurrences of this phenomenon and their relevance are unknown.

The combination of the previous considerations may result in significantly high impedance values for the frequency range assigned to BPL. As a result, the impedance mismatching can be relevant at specific frequencies, and the propagation of the PLC signals may be severely conditioned. Moreover, the resonance effects may cause remarkable variations of the impedance values for specific frequency slots. A detailed characterization of the grid impedance in both time and frequency domains is necessary to determine the propagation conditions of the BPL signals. This characterization requires extensive field trials in different grid topologies and types of connected loads.

### 7.3. Noise and Non-Intentional Emissions (NIEs)

The electrical grid is facing a transformation to a decentralized scheme, where the consumer has an active role, and power generation is evolving from central to distributed networks. This evolution is incorporating new techniques to generate and store energy, such as different techniques employing renewable energy resources, electrical vehicle charging points, and small battery chargers. Most of these devices are based on power electronic converters, which generate changes in the grid impedance and high levels of conducted noise and emissions that are injected into the distribution grid. Hence, the deployment of a higher number of electronic devices connected to the grid, in conjunction with improvements in power electronics for energy efficiency, are increasing the number and the levels of conducted emissions in the frequency bands assigned to PLC [121,123].

According to [139,140,141], the NIEs of the Low Voltage (LV) distribution grid are composed of background and impulsive noise. The background noise consists of colored noise, caused by home appliances and a low Power Spectral Density (PSD), and narrowband interferences, which are mainly due to broadcast radio bands. The impulsive noise can originate from the rectifier diodes included in the circuitry of the power supplies, which implies a periodic noise synchronous with the mains cycle. In other cases, also due to switching power supplies, periodic impulsive noise asynchronous with the mains signal and repetition rates between 50 kHz and 200 kHz can be found [142]. Finally, aperiodic impulsive noise, as a consequence of the connection or disconnection of electrical devices, may also be present in the grid. Some techniques and models for analyzing the grid noise have been reported in the literature. In [143,144,145,146], a statistical characterization of the impulsive noise is carried out, whereas in [140,141], the background noise is modeled.

In frequencies over 1 MHz, the level of noise and NIE are still unknown, along with the distance they propagate thorough the grid and therefore the disturbance they may cause in the broadband communication devices. Accordingly, only some deductions from the results in lower frequencies can be derived.

Different studies have demonstrated that in some cases, the NIEs are causing a relevant disturbance in the deployed communication technologies up to 150 kHz (NB-PLC) [2,121,122,147,148,149,150]. In particular, voltage levels above the compatibility levels defined in [151] increase the risk of interference for some forms of equipment connected to the grid. Furthermore, as the NIEs spread through the grid to neighboring locations [152], the scope of the impact on the communications increases, and communication devices located at a certain distance may also be affected [121,147,149]. The significance of the impact depends on the level, the spectral shape, and the time variation of the NIE [121,123,149].

Some recently developed measurement campaigns in the LV grid have identified the more relevant NIE sources and the NIE characteristics for frequencies below 150 kHz. The highest-level emissions are usually generated by electronic devices based on inverters, such as EV chargers, PV panels, battery chargers, power supplies, elevators, or engine control systems, which generate a set of high-level narrowband emissions at multiple frequencies of the switching frequency of the inverter [122,123,149,150,153,154,155]. The switching frequency of these devices greatly depends on the application, but current technologies tend to use significantly higher frequencies. According to [156,157], switching frequencies from 20 kHz to 300 kHz are currently used for both high-power and low-power applications. The increasing number of such devices leads to more frequent and higher levels of emissions in the frequency range from 2 kHz–150 kHz, as they operate with switching frequencies of several kHz [123,149,153]. Many of these interfering devices are located close to SMs, as in the case of EV chargers and PV panel inverters, increasing the possibility of affecting the performance of the communications. Other sources, such as motors [153], lighting devices [158,159,160], or electronic amplifiers [122], cause high-level noise in a wide frequency range, without specific spectral patterns. Additionally, the devices mentioned above may generate impulsive emissions of considerable amplitude when they commute between different states or change the working regimes [153]. Lastly, as the NIE present in the grid are an ensemble of the emissions generated by different sources, the amplitude and the frequencies of this combination vary with time, because they depend on the type, the number and the working conditions of the devices connected to the grid at each moment [147,148,149,150]. The NIEs that these devices may generate in frequencies above 1 MHz is unknown.

The field measurements up to 500 kHz show that the amplitude of the noise and NIEs decrease with frequency in most of the measurement points, although this tendency is not fulfilled in all of them [153,154]. In general, the highest levels of noise and conducted emissions are found in frequencies up to 50 kHz; from 50 kHz to 150 kHz, the amplitudes of the NIEs vary, depending on the nearby sources; in the range from 150 kHz–500 kHz, the level is, in general, considerably lower. According to this tendency, it is expected that the NIEs show decreasing amplitudes for frequencies above 500 kHz, which means a less interfering environment for communications. This is a strong point in favor of the use of frequencies above 500 kHz for PLC.

Nevertheless, the impact of DERs and EV chargers is one of the main concerns in this area, due to the increasing density expected in the coming years, and the negative effects caused by the combination of their emissions on the surrounding communication devices in charge of transmitting consumption and mains quality data. The accumulative effects of the NIEs generated by a high number of these electronic devices in a relatively small area is a matter to be analyzed; the results of this study are relevant to determining the overall impact of a fast deployment of EV chargers and DERs on the performance of BPL technologies. Due to the wide variety of noise sources, the lack of field data, and the unknown propagation properties of the grid for higher frequencies, a detailed characterization and quantification of this phenomenon must be developed. This study should be based on field measurements developed in representative SG scenarios and grid topologies.

One last aspect of this issue is the lack of standardization for frequencies above 2 kHz, in particular about the limits of emission, compatibility, and immunity [2,123,161], and also about the standardized measurement methods [162]. The standardization and regulatory bodies have made a great effort in the last few years to generate updated regulation for frequencies up to 150 kHz, launching working groups and task forces in different areas: CENELEC SC205 (currently, TC219) [121], IEC SC 77A group [163], IEEE EMC Society TC7 group [164], IEEE P1250 (Power and Energy Society) [165], and the CIGRE-CIRED working group C4.24 [166]. The work in the frequency range from 150 kHz–500 kHz is more limited, and only CENELEC TC219 is preparing a report about this topic. For frequencies above 500 kHz, there is still a long way to go. A representative example of this lack of regulation is that the limits of NIE from DERs and other types of equipment have not been established for the frequencies assigned to PLC [2].

### 7.4. Channel Modelling and Transmission Losses

The transmission losses of the signal propagated through the electrical grid will certainly be one of the critical aspects in deploying BPL technologies in the LV distribution grid [124]. Some studies concerning ranges up to 500 kHz have demonstrated that the transmission losses caused by the electrical cables increase at higher frequencies [167,168]. Moreover, the splits in the distribution grid into different branches generate additional losses in the data transmission, which are difficult to determine with accuracy and are therefore difficult to include in mathematical models. On a broader scale, other factors are involved in the signal attenuation of the propagated signal, such as cable type, frequency, the coupling method of PLC devices, the line length of different sections of the distribution, the total line length, and the terminating impedances [168]. The influence of these factors in the significance of the transmission losses is still to be analyzed.

As stated in [169], the characterization and modelling of the PLC channel can be performed by means of two different approaches: the top-down approach and the bottom-up approach. The top-down approach is based on a large number of measurements where the channel is excited by a reference signal in the time or frequency domain. This approach has been considered in several published works [170,171]. In addition, the MIMO PLC channel has also been modeled by applying this approach, as included in [172,173]. In contrast, the bottom-up approach is focused on modelling the channel by applying the transmission line theory [174]. Hence, the two-conductor line (2TL) theory is employed to power grids connected with two-conductor transmission lines; some studies regarding this model can be found in [175,176,177,178,179]. Some other works, such as [180,181,182,183,184,185,186], consider the multi-conductor transmission line (MTL) model as a generalization of the already mentioned 2TL approach. This method is also presented in [187] for the modelling of the MIMO PLC channel.

Some authors have proposed different channel models to assess the propagation effects of the electrical grid at higher frequencies. For example, in [188], it is proposed that attenuation increases linearly with frequency, and as a result, it can be represented as a straight line with a positive slope, so that a simple equation can be used to calculate the amplitude of the channel transfer function. Nevertheless, this approach might be too simple for many cases, as it does not consider effects such as multipath propagation [167] or resonance effects.

Some multipath propagation approaches can be found in the literature. In [189], the channel transfer function is defined as the direct superposition of N paths of different amplitudes and delays, which provides a closer representation of the resulting notches generated by the delayed echoes that compose the multipath. This model may be representative of indoor channels, where the multipath is one of the most relevant effects. Still, it does not consider other effects present in the distribution grid, such as the splits of the grid or resonance effects.

Zimmerman includes an additional frequency-dependent attenuation factor, which is applied to every component of the multipath [190], and it leads to a model channel that can be similar to the behavior of the electrical grid, as it differs with the frequency range. Therefore, this model more flexibly represents two critical effects in the propagation channel of the electrical grid: the frequency-dependent attenuation and the presence of frequency-selective fading [167].

An empirical model based on measurements performed by the OPERA project [191] is derived in [137]. According to this proposal, the path losses in the LV grid increase dramatically with frequency for frequencies above 1 MHz, with attenuation values higher than 50 dB for frequencies over 10 MHz. The high number of branches in the LV distribution grid, together with the high resistance values generated at high frequencies by the parallel resonance of the close three-phase cables of the LV grid, seem to be the crucial aspects [137].

If the effects mentioned above are extrapolated to frequencies assigned to BPL (1 MHz–30 MHz), great attenuation values are expected, mainly for the upper part of the spectrum. Considering that the attenuation found for frequencies up to 500 kHz are, in many cases, higher than 30 dB between adjacent communication devices [192], the attenuation above 1 MHz could exceed 60–80 dB between the SMs located in the homes and the DC in the TC, depending on the grid topology [137,167]. As a result, for grid sections formed by a high number of branches and long cable sections (>200 m), the communication between adjacent devices could be limited in frequency to a few MHz [167]. It is important to mention that these considerable transmission losses for the grid cannot be compensated by a higher transmitted signal power due to electromagnetic compatibility limitations. This noticeable limitation may be overcome with the use of coding techniques that have demonstrated enhanced robustness, such as LDPC codes [193], at the expense of reducing the net data rate and increasing the complexity of the communication devices.

Unfortunately, the lack of results from field trials at frequencies above 1 MHz avoid the estimation of the grid attenuation, and as a result, the assessment of the maximum distance between adjacent transmission devices where BPL links are feasible. This is a basic aspect to plan the deployment of the communication network, and potentially, the needs of devices operating as relays or gateways.

Therefore, extensive field trials in representative scenarios and use cases are necessary, firstly, to characterize the grid frequency-dependent attenuation; secondly, to determine the frequency ranges where successful data transmissions can be reached; and lastly, to assess the channel capacity that can be achieved. Based on this information, the adaptation of grid assets might be necessary for the creation of PLC signal repetition opportunities in the locations where an excess of attenuation does not allow proper signal propagation.

### 7.5. Cybersecurity

The electrical grid is a huge network spread across urban and rural areas to reach every home with a critical service. It is an exposed infrastructure, also used for private data transmission related to Smart Grid services. In this context, the security of the grid is necessary, mainly in the following aspects:

- The propagated data related to the users’ consumption are sensitive as they provide private information about the use patterns and the periods when users are out of the home.
- The data about the grid power quality and the state of the network are sensitive data for the utility.

As the power grid becomes increasingly digital, its vulnerability to cyberattacks grows, since the number of connected devices increases alongside the data traffic. With the proliferation of smart metering systems across the Smart Grid, the amount of information transmitted through the grid increases considerably, and consequently, the relevance of integrity and confidentiality becomes progressively greater. Moreover, connected equipment such as Industrial Control Systems (ICS) and SCADA networks are just two of the many common IT assets increasingly present in power systems, meaning that the vulnerabilities inherent to these systems and their risks for cyber-attacks can affect the global performance of the energy system. The benefits of the decentralized management of assets and resources in the power grid, with their corresponding sensing and control systems, are indeed a considerable drawback when dealing with cybersecurity. As a result, while digitalization grows in the energy sector, cybersecurity becomes a major issue.

The security issues can be external or internal, as both eavesdropping or transmission of the frames can be carried out by a legitimate user connected to the grid or by an external third party [124,194]. In any case, both data protection and non-access to non-authorized third parties must be ensured.

From a cybersecurity perspective, a key element in the power grid architecture is the smart meter, as it can be a sensitive point in two ways. Firstly, smart metersmanage a massive amount of data that may be sensitive, which could raise both privacy and security concerns if not properly protected. Secondly, smart meters entail an entry point to the network, increasing the vulnerability to attacks.

Some studies have analyzed the Physical Layer Security (PLS) of the already deployed PLC and PLC/wireless technologies. According to the theoretical results, higher secrecy rates are provided by wireless fading channels in comparison with the PLC channels [195], while the use of hybrid PLC/wireless technologies can provide increased secrecy for low-bit-rate applications [196], mainly if coding diversity is applied [197].

The security issues regarding ITU-T G.hn BPL for access networks are being discussed and include a set of improvements that can be added to the standard to deal with stricter requirements. There is also concern regarding the spread of IoT systems in the power grid, since poorly protected IoT devices could be used for harmful purposes.

The use of a new frequency range for BPL to transmit higher data rates for new Smart Grid services must be accompanied by robust cybersecurity techniques and encryption algorithms that hinder the access to the data and detect attempted attacks. The security issues regarding the current implementations of ITU-T G.hn BPL for access networks are discussed in [194]. The study shows that the authentication procedure AES-128 defined in the physical layer of G.hn cannot grant authenticity, since PLC credentials are “public” by definition. Therefore, it proposes three security concepts in different complexities to adapt the key features of IEEE 802.1X into G.hn: the encryption of the authentication messages during the handshake, including additional response messages and the insertion of Extensible Authentication Protocol—Transport Layer Security (EAP-TLS) authentication to validate the possession of the private keys.

Lastly, it must be considered that for frequencies above 1 MHz, the portion of the propagated signals radiated by the cable is higher than for frequencies used in NB-PLC, mainly if it is poorly shielded. This phenomenon hinders the security protection in the physical layer of BPL, as a third party could access the signals without accessing the cable.

The mitigation techniques are based on encrypting lower layers of the protocol stack [2], by enhancing the robustness against this type of vulnerabilities, at the expense of reducing the net data rate that can be achieved. In addition, techniques explored for NB-PLC, such as using physical layer keys to encode the data, known only by the two communication ends [198], or securing a DLMS/COSEM protocol [199], could be applied to BPL technologies.

## 8. Conclusions

In this article, a comprehensive review of the original and the state-of-the-art BPL transmission technologies has been provided. First, the main organizations, technological alliances, and standardization institutions involved in the development of BPL technologies have been introduced. Then, the fundamental characteristics of the physical and medium access control layers of different technologies were gathered and compared. The regulatory issues related to the conducted and radiated emissions that BPL systems must comply with have been summarized. Finally, some representative applications and use cases have been introduced, together with the challenges that future applications based on BPL technologies should face to be successful, namely, the grid access impedance, the non-intentional emissions, the transmission losses, and cybersecurity issues. The time and frequency-dependent characteristics of the medium, mainly due to the fact that power line cables were not originally designed for data communication, can considerably affect the proper development of BPL. Therefore, the characterization of the electrical grid as a transmission medium and the adaptation of these technologies to this harsh communication channel, are instrumental aspects in the deployment of BPL.

BPL communications are enlisted to play an important role in the development of new Smart Grid services and applications, due to the high-performance features that will be demanded in the transformation of the electrical grid model in the next few years.

BPL communications are the most immediate and affordable answer to the SG challenge in the distribution grid. While Smart Metering has been instrumental to reaching the outer edge of the grid (i.e., the customer), the capability to deploy broadband communications through the grid is not just a question of the natural evolution of telecommunication technologies but is a requirement for accomplishing the SG evolution. In particular, BPL technologies are a key tool for the control and monitoring of secondary substations. They will also be essential in the distribution grid, in terms of PQ monitoring and distribution generation, especially considering the growth of solar and wind power sources and the progressive introduction of massive EV fleets, with hundreds of vehicles charging and discharging at the same time [200]. In addition, BPL technologies have a great potential towards the diagnosis of the power grid and security assurance. The methods for cable health monitoring are mainly based on the topological parameters of the network, physical properties of the cable, and the measured QoS parameters, while other methods include simulations and diagnostics solutions [201].

Compared to other telecommunication alternatives, BPL deployment is not driven by the wider telecommunications commercial market, but it has its own dynamics. They remain available for underground operation, as required in some underground transformer stations, but unlike optical fiber solutions, BPL communications do not require great investments due to a new optical fiber-based network. Specifically in the LV distribution grid, the BPL technologies enable enhanced grid monitoring, real-time automation, and sophisticated customer and grid related energy programs, which are key aspects of the SG. However, there are also some technical constraints that need to be faced, such as high transmission losses, a wide range of grid impedance values, and radiation limits.

The success of BPL technologies requires a solid roadmap, which entails, firstly, a standardization work that allows the BPL technologies to keep improving and adapting for the upcoming challenges; secondly, interoperability among BPL technologies, as well as some compatibility with other PLC technologies already worldwide deployed (e.g., NB-PLC); and lastly, strong industrial and utilities alliances that support the development of BPL products and their rollouts.

## Figures and Tables

**Table 1:** Comparison of technical specifications of main BPL technologies.

Technology		Data Rate	Frequency Band	Codes	Modulations	Media Access Control
G.hn	G.hn LCP	5–20 Mbps	2–25 MHz	LDPC	FFT-OFDM/QPSK	TDMA
	G.hn	1 Gbps	2–100 MHz		FFT-OFDM/QAM	
IEEE 1901	2010 FFT	>100 Mbps	1.8–50 MHz	Turbo Convolutional code	FFT-OFDM/BPSK, QPSK, 8 QAM, 16 QAM, 32 QAM, 64 QAM, 1024 QAM, 4096 QAM	TDMA, CSMA/CA
	2010 Single Channel Wavelet (Baseband)	>100 Mbps	1.8–28 MHz (optional 30–50 MHz)	Reed–Solomon, Convolutional code (Viterbi), LDPC	Wavelet-OFDM/2-PAM, 4-PAM, 8-PAM, 16-PAM, 32-PAM (high-speed mode)	
					Wavelet-OFDM/2-PAM (diversity mode)	
	2010 Single Channel Wavelet (Bandpass)	>100 Mbps	1.8–50 MHz	Reed–Solomon, Convolutional code (Viterbi), LDPC	Wavelet-OFDM/2-PAM, 4-PAM, 8-PAM, 16-PAM, 32-PAM (high-speed mode)	
					Wavelet-OFDM/2-PAM (diversity mode)	
	2020 Flexible Channel Wavelet	>100 Mbps	1.8–28 MHz (optional 31.25–62.5 MHz)	Reed–Solomon, Convolutional code (Viterbi), LDPC	Wavelet-OFDM/2-PAM, 4-PAM, 8-PAM, 16-PAM, 32-PAM (high-speed mode)	
					Wavelet-OFDM/2-PAM (diversity mode)	
HD-PLC	1st gen.	190 Mbps	4–28 MHz	Reed–Solomon, Convolutional code (Viterbi), LDPC	Wavelet-OFDM	CSMA/CA
	2nd gen.	210 Mbps	2–28 MHz			
	IEEE 1901–2010 (3rd gen. Complete)	240 Mbps	2–28 MHz			
	ITU-T G.9905 (3rd gen. Multi-hop)	240 Mbps	2–28 MHz			
	IEEE 1901–2020 (4th gen.)	1 Gbps	2–100 MHz			
HomePlug	1.0	14 Mbps	4.5–21 MHz	Viterbi Reed–Solomon	FFT-OFDM/DBPSK, DQPSK	CSMA/CA
	AV 1.0	200 Mbps	2–28 MHz	Turbo Convolutional Codes	FFT-OFDM/DBPSK, DQPSK, 16 QAM, 64 QAM, 256 AM, 1024 QAM	TDMA, CSMA/CA
	AV 1.1	200 Mbps				
	AV 2.0	1.5 Gbps	1.8–86.3 MHz	Turbo Convolutional Codes	FFT-OFDM/DBPSK, QPSK, 16 QAM, 64 QAM, 256 QAM, 1024 QAM, 4096 QAM	TDMA, CSMA/CA
	Green PHY	10 Mbps	2–30 MHz	Turbo Codes	FFT-OFDM/QPSK	CSMA/CA
OPERA		200 Mbps	2–30 MHz	Reed–Solomon	OFDM/ADPSK Truncated four-dimensional Trellis coded	TDMA
KS X 4600-1	Class A	24 Mbps	2.15–23.15 MHz	Reed–Solomon Convolutional Coding	DBPSK, DQPSK, D8PSK	CSMA/CA
	Class B	200 Mbps				

**Table 2:** General class B limits for conducted disturbances.

Frequency Range (MHz)	Limits (dBµV)	
	Quasi-Peak	Average
0.15 to 0.50	66 to 56	56 to 46
0.50 to 5	56	46
5 to 30	60	50

NOTE 1: The lower limit applies at the transition frequencies. NOTE 2: The limit decreases linearly with the logarithm of the frequency in the range 0.15 MHz to 0.50 MHz.

**Table 3:** General class B limits for radiated disturbances.

Frequency Range (MHz)	Measurement			Class B Limits (dBµV/m)
	Equipment	Distance (m)	Type of Detector/Bandwidth	
30 to 230	OATS/SAC	10	Quasi-peak/120 kHz	30
230 to 1000				37
30 to 230	OATS/SAC	3		40
230 to 1000				47
30 to 230	FAR	10	Quasi-peak/120 kHz	32 a 25
230 to 1000				32
30 to 230	FAR	3		42 a 35
230 to 1000				42

**Table 4:** Maximum PLC transmit signal level between 1.6065 MHz and 30 MHz.

Symmetrical Mode Insertion Loss EUT to AE in dB	10	20	≥40
Maximum transmit signal level in dB (μV) (AV)	65	75	95
Maximum transmit signal level in dB (μV) (PK)	75	85	105

NOTE: The transmitted power management function of an EA should work in the same way as that of the ESE. Otherwise, the EA signal may dominate and cause erroneous results during the measurement.
